# HIV/AIDS in Nigeria: a bibliometric analysis

**DOI:** 10.1186/1471-2334-8-19

**Published:** 2008-02-26

**Authors:** Olalekan A Uthman

**Affiliations:** 1 Center for Evidence-Based Global Health, Save the Youth Initiative, Nigeria

## Abstract

**Background:**

Nigeria is home to more people living with HIV than any other country in the world, except South Africa and India-where an estimated 2.9 million [1.7 million – 4.2 million] people were living with the virus in 2005. A systematic assessment of recent HIV/AIDS research output from Nigeria is not available. Without objective information about the current deficiencies and strengths in the HIV research output from Nigeria, it is difficult to plan substantial improvements in HIV/AIDS research that could enhance population health. The aim of this study was to analyse the trends in Nigeria's SCI publications in HIV/AIDS from 1980 to 2006. Special attention was paid to internationally collaborated works that were identified based on the countries of the authors' affiliation.

**Methods:**

A bibliometric analysis regarding Nigerian HIV/AIDS research was conducted in the ISI databases for the period of 1980 to 2006. An attempt was made to identify the patterns of the growth in HIV/AIDS literature, as well as type of document published, authorship, institutional affiliations of authors, and subject content. International collaboration was deemed to exist in an article if any co-author's affiliation was located outside Nigeria. The impact factors in the 2006 Journal Citations Reports Science Edition was arbitrarily adopted to estimate the quality of articles.

**Results:**

Nigeria's ISI publications in HIV/AIDS increased from one articles in 1987 to 33 in 2006, and the articles with international collaboration increased from one articles in 1980 to 16 in 2006. Articles with international collaboration appeared in journals with higher impact factors and received more citations. A high pattern of co-authorship was found. Over 85% of the articles were published in collaboration among two or more authors. The USA, as the most important collaborating partner of Nigeria's HIV/AIDS researchers, contributed 30.8% of articles with international collaboration.

**Conclusion:**

Nigeria has achieved a significant increase in the number of SCI publications and collaborations in HIV literature from 1987 to 2005. There is need to challenge the status, scientists from Nigeria should forge multiple collaborations beyond historical, political, and cultural lines to share knowledge and expertise on HIV/AIDS.

## Background

Nigeria is home to more people living with HIV than any other country in the world, except South Africa and India-where an estimated 2.9 million [1.7 million – 4.2 million] people were living with the virus in 2005 [1]. One vital element in improving this situation is the need for a comprehensive and relevant evidence base that would equip Nigeria to take informed actions. A systematic assessment of recent HIV/AIDS research output from Nigeria is not available. Without objective information about the current deficiencies and strengths in the HIV research output from Nigeria, it is difficult to plan substantial improvements in HIV/AIDS research that could enhance population health. Bibliometrics has a long tradition as the preferred method of choice for quantitative assessments of academic research on national, institutional, and individual levels [2-5]. Bibliometric analysis has also been performed within and across the above mentioned levels in the evaluation of research areas [6-9]. The aim of this study was to analyse the trends in Nigeria's SCI publications in HIV/AIDS from 1980 to 2006. Special attention was paid to internationally collaborated works that were identified based on the countries of the authors' affiliation.

## Methods

### Data sources

From the Web of Science^® ^(including SCI Expanded, Social Sciences Citation Index, and Arts Humanities Citation Index) on the Internet [10], records indexed to HIV/AIDS and belonging to Nigerian based on the addresses of the authors' affiliations were downloaded. The period of analysis was limited to the publication years between 1980 and 2006. Additional records were retrieved by searching for major cities and all states in Nigeria using search terms and strategy described in Table 1.

**Table 1:** Search strategy

**Number**	**Search terms**
No 1	PS = (Abia OR Adamawa OR Akwa Ibom OR Anambra OR Bauchi OR Bayelsa OR Benue OR Borno OR Cross River OR Delta OR Ebonyi OR Edo OR Ekiti OR Enugu OR Abuja OR Gombe OR Imo OR Jigawa OR Kaduna OR Kano OR Katsina OR Kebbi OR Kogi OR Kwara OR Lagos OR Nassarawa OR Niger OR Ogun OR Ondo OR Osun OR Oyo OR Plateau OR Rivers OR Sokoto OR Taraba OR Yobe OR Zamfara)
No 2	CI = (Umuahia OR Yola OR Uyo OR Awka OR Bauchi OR Yenagoa OR Makurdi OR Maiduguri OR Calabar OR Asaba OR Abakaliki OR Benin City OR Ado-Ekiti OR Enugu OR Abuja OR Gombe OR Owerri OR Dutse OR Kaduna OR Kano OR Katsina OR Birnin Kebbi OR Lokoja OR Ilorin OR Ikeja OR Lafia OR Minna OR Abeokuta OR Akure OR Oshogbo OR Ibadan OR Jos OR Port Harcourt OR Sokoto OR Jalingo OR Damaturu OR Gusau)
No 3	CU = (NIGERIA)
No 4	No 1 OR No 2 OR No 3
No 5	TS = (HIV Infections OR HIV OR hiv-1* OR hiv-2* OR hiv1 OR hiv2 OR hiv infect* OR human immunodeficiency virus OR human immunedeficiency virus OR human immuno-deficiency virus OR human immune-deficiency virus OR acquired immunodeficiency syndrome OR acquired immunedeficiency syndrome OR acquired immuno-deficiency syndrome OR acquired immune-deficiency syndrome OR AIDS)
No 6	TI = (HIV Infections OR HIV OR hiv-1* OR hiv-2* OR hiv1 OR hiv2 OR hiv infect* OR human immunodeficiency virus OR human immunedeficiency virus OR human immuno-deficiency virus OR human immune-deficiency virus OR acquired immunodeficiency syndrome OR acquired immunedeficiency syndrome OR acquired immuno-deficiency syndrome OR acquired immune-deficiency syndrome OR AIDS)
No 7	No 5 OR No 6
No 8	No 4 AND No 7
No 9	No 8 LIMITS: DocType = Article OR Review; Language = All languages; Year = 1980 to 2006

The impact factors of 6, 164 journals listed in the 2006 Journal Citation Reports Science Edition^® ^[11] were arbitrarily adopted to estimate the quality of articles. Analysis was limited to the "articles" and reviews papers indexed to HIV/AIDS in the ISI database. Notes, letters, editorials, news and meeting abstracts were excluded. Authors' affiliations and countries were identified from the fields of affiliation and corresponding address. International collaboration was deemed to exist in an article if any co-author's affiliation was located outside Nigeria. Publication counts and the share of articles with international collaboration in each year was computed. The data were then stratified by journal impact factor, subject category, domestic institution and collaborating country. To accredit an article to institutions and countries, method of "absolute country counting" was adopted, in which each institution or country contributing to an article received one paper credit, respectively [12].

### Statistical analysis

Besides the descriptive statistics (e.g. the frequency in count and percentage), the associations between number of articles and the share of articles with international collaboration in each year; and between the article count of the subject categories and their share of articles with international collaboration was also computed using Kendall's tau-b correlation coefficient. Difference in number of citations received by an article and the impact factor of the journal in which an article was published between (1) group of articles with international collaboration and articles without international collaboration, (2) articles with four or more authors and articles with fewer than four authors were compared using Mann-Whitney U test. The cut-off points for number of authors is based on the median value. A p value < 0.05 was regarded as statistically significant (2-tailed). Statistical analyses were performed using Stata 10.0 for Windows [13].

## Results

### Production and authorship

**Figure 1:**
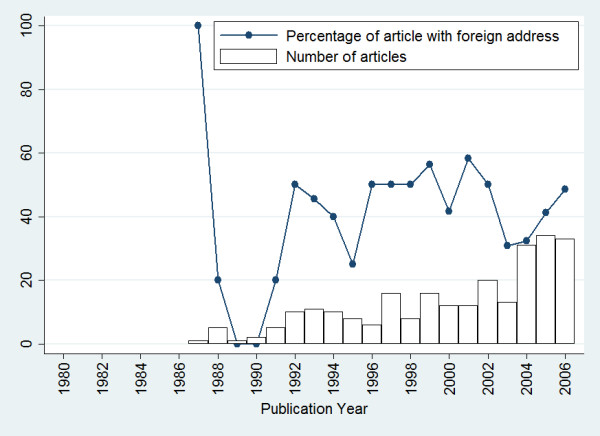
**Trends in Nigeria's HIV/AIDS literature publications in Web of Science**. No records were found for the period of 1980 to 1986.

A total of 254 records of articles in which the publication years were between 1987 and 2006, indexed to HIV/AIDS, and at least one author's affiliation was located in Nigeria. Most of the studies [n = 253 (99.6%)] were in English language. Only one article was published in French. Nigeria's ISI publications in HIV/AIDS increased from one articles in 1987 to 33 in 2006, and the articles with international collaboration increased from one articles in 1980 to 16 articles in 2006. No records were found for the period of 1980 to 1986; and only one record was retrieved for 1987 and 1989 (Figure 1). There was not statistically significant correlation between number of articles in each year and percentage of articles with international collaboration (Kendall's tau-b coefficient = 0.23, p = 0.139).

**Figure 2:**
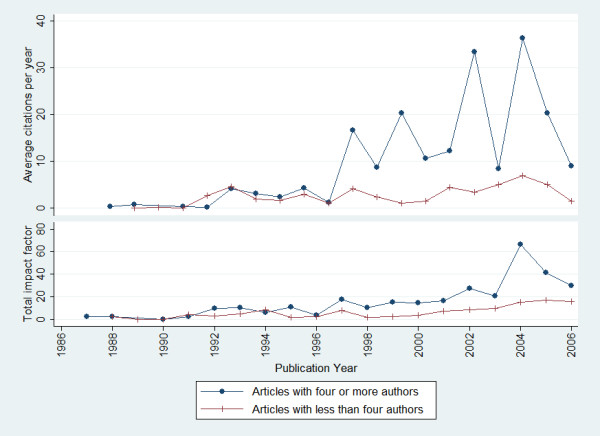
**Citation performance of articles with four or more authors and articles with less than four authors**. The figures for more recent years are lower because papers published during this period have had less time to accumulate citations.

84.2% of the publications were co-authored. The median number of authors was 4 (range, 1 to 27). Table 2 provides a distribution of the number of authors per publication. On average, articles with four or more authors appeared in journals with higher impact factors than those fewer than four authors (median: 1.1 vs. 2.1; P < 0.0001, Mann-Whitney U test). Similarly, articles with four or more authors also received more citations (median: 1.5 vs. 5.0; P < 0.0001, Mann-Whitney U test) (Figure 2).

**Table 2:** Distribution of the number of authors per publication

Number of authors	Number of publication	Percent
1	40	15.8
2 – 5	138	54.3
6 – 10	55	21.6
11 – 15	16	6.3
16	5	2.0

Total	254	100.00

### Journal impact factors and citations

**Figure 3:**
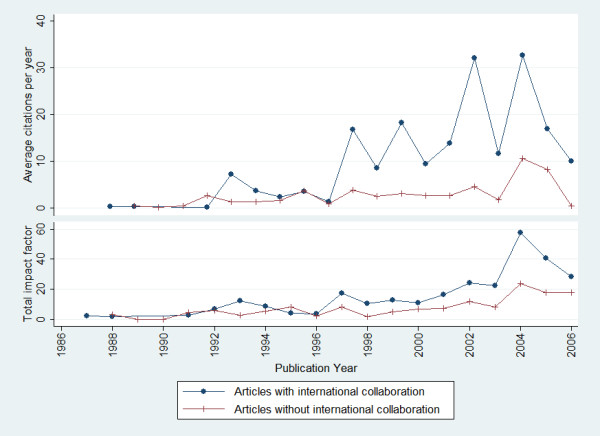
**Citation performance of articles with or without international collaboration**. The figures for more recent years are lower because papers published during this period have had less time to accumulate citations.

Based on the journal listed on the journal impact factors in 2006, only 17 (6.7%) articles were published in journals with impact factor equal to or greater than five (Table 3). The probability of international collaboration increased with the journal impact factors: 100.0% in articles with a higher journal impact factor in contrast with 19.7% in articles with a journal impact factor less than one. Figure 3 provides a comparison between the impact factors and average citation per year for papers with authors from Nigeria alone and papers with papers with foreign address. The figures for more recent years are lower because papers published during this period have had less time to accumulate citations. On average, articles with international collaboration appeared in journals with higher impact factors than those without international collaboration (median: 1.078 vs. 2.476; P < 0.0001, Mann-Whitney U test). Similarly, articles with international collaboration also received more citations (median: 1.0 vs. 6.0; P < 0.0001, Mann-Whitney U test).

**Table 3:** Distribution of Nigeria's HIV/AIDS publications in the Web of Science by journal impact factors

Journal impact factor*	No. of all articles	Articles with foreign address, n(%)
≥ 5	17	17 (100.0)
≥ 4 and < 5	2	0 (0.0)
≥ 3 and < 4	14	11 (78.6)
≥ 2 and < 3	54	36 (66.7)
≥ 1 and < 2	55	17 (30.9)
< 1	66	13 (19.7)
NA	46	15 (31.3)

Total	254	109 (42.9)

*The journal impact factor was based on the 2006 Journal Citation Reports Science Edition, NA = Not available

### Institutions

In Table 4, the top 20 Nigerian institutions in HIV/AIDS publications from 1987 to 2006 are shown. University of Ibadan (including University College Hospital, Ibadan) obtained the highest frequency, with 47 records, followed by University of Lagos (27 records). Over 77% of the records included a corporate source. An analysis of these records revealed that the main participating authors were from United States (61), United Kingdom (24), and South Africa (12) (Table 5). The USA, as the most important collaborating partner of Nigeria's HIV/AIDS researchers, contributed 24.0% of the total articles. Centers for Disease Control and Prevention, Atlanta and Harvard University in obtained the highest frequency with nine records each, followed by University of Maryland, Maryland, with seven records. Table 6 provides a list of the main United States institutions found.

**Table 4:** Distribution of Nigeria's HIV/AIDS publications in the Web of Science by Nigerian institutions (selected)

Institution Name	Record Count	Percent
University of Ibadan	27	10.5
University Lagos	27	10.5
Obafemi Awolowo University	19	7.4
University Calabar	19	7.4
University Jos	18	7.0
University Maiduguri	20	7.5
University Nigeria Nsukka	18	7.0
University College Ibadan Hospital	20	7.5
University Benin	14	5.4
Federal Ministry of Health	10	3.9
Nigerian Institute of Medical Research	9	3.5
University Ilorin	9	3.5
Ahmadu Bello University	4	1.6
Ondo State University	4	1.6
University Port Harcourt	3	1.2
Ahmadu Bello University Hosp	2	0.8
Aminu Kano Teaching Hosp	2	0.8
Chidak Med Diagnost Labs	2	0.8
Nnamdi Azikiwe University	2	0.8

Total	229	90.2

Note: Percentages may not add up to 100% because only institutions with atleast two articles are list

**Table 5:** Distribution of Nigeria's HIV/AIDS publications with international collaboration in the Web of Science by collaborating (selected)

Country	Number of publications	Percent
United States	61	24.0
England	24	9.4
South Africa	12	4.7
France	10	3.9
Australia	8	3.1
Cameroon	7	2.8
Canada	7	2.8
Zimbabwe	6	2.4
Belgium	5	2.0
India	5	2.0
Japan	5	2.0
Niger	5	2.0
Mali	4	1.6

Total	159	62.5

Note: Percentages may not add up to 100% because only countries with more than three articles are list

**Table 6:** Distribution of Nigeria's HIV/AIDS publications in the Web of Science by United States institutions

Institution Name	Record Count	Percent
Centers for Disease Control Prevention	9	3.5
Harvard University	9	3.5
University Maryland	7	2.7
National Institute of Pharmaceutical Research and Development	6	2.3
Johns Hopkins University	5	2.0
National Cancer Institute	5	2.0
University Texas	5	2.0
University South California	4	1.6
University Western Ontario	4	1.6
National Institute of Allergy and Infectious Diseases	3	1.2
Columbia University	2	0.8
Family Health International	2	0.8
University of Michigan	2	0.8

Total	61	24.0

### Subject category and Source titles

All articles were indexed into 84 subject categories. Table 7 displays 28 subjects' categories with three or more records. Of the 254 records indexed with the description of HIV/AIDS, 75 were also indexed with subject category Public, environmental and occupational health, 43 with infectious diseases, 36 with immunology, 34 with tropical medicine, and 27 with virology. There was positive correlation between number of articles in each subject categories and their percentage of articles with international collaboration (Kendall's tau-b coefficient = 0.112 p = 0.001). A total of 135 periodical titles were found. East African Medical Journal and Tropical Doctor were the most frequent title, with 13 postings each, followed by AIDS Care (12), AIDS (8), AIDS research and Human Retroviruses (7), International Journal of Gynecology and Obstetrics (7), and International Journal of STD and AIDS (7). These seven periodicals covered quarter of the total production.

**Table 7:** Distribution of Nigeria's HIV/AIDS publications in the Web of Science by subject category (selected)

Subject category	Number of articles	Percent*
Public, environmental & occupational health	75	29.2
Infectious diseases	43	16.7
Immunology	36	14.0
Tropical medicine	34	13.2
Virology	27	10.5
Medicine, general & internal	26	10.1
Social sciences, biomedical	22	8.6
Health policy & services	16	6.2
Psychology, multidisciplinary	12	4.7
Pediatrics	11	4.3
Microbiology	8	3.1
Demography	8	3.1
Biotechnology & applied microbiology	8	3.1
Obstetrics & gynecology	7	2.7
Dermatology	7	2.7
Medicine, research & experimental	6	2.3
Dentistry, oral surgery & medicine	6	2.3
Multidisciplinary sciences	5	1.9
Family studies	5	1.9
Education & educational research	5	1.9
Pharmacology & pharmacy	4	1.6
Surgery	4	1.6
Hematology	4	1.6
Medical laboratory technology	3	1.2
Veterinary sciences	3	1.2
Health care sciences & services	3	1.2
Nursing	3	1.2
Parasitology	3	1.2

Total*	394	155.1

*Note: Some articles are indexed with several subject categories

## Discussion

### Main findings

This study was a purely descriptive analysis about Nigeria's SCI publications in HIV/AIDS research for over two decades and arbitrarily adopted co-authorship as an indicator of collaboration. The results showed a continuous increases and reassuring trends in the number of HIV literature production from Nigeria. However, no records were found for period between 1980 and 1986. This finding is consistent with data from other study [14] that has examined this variable. However, the finding is not consistent with those of others [15-17]. Huber [15] found no records in AIDSLINE, EMBASE, CINAHL, and MEDLINE for the years 1980 through 1982. Macias-Chapula [16] found no records for the period between 1980 and 1981. Pratt [17] reported in 1992 that AIDS literature in MEDLINE grew from fewer than 700 entries for the period 1981 through 1983 to a cumulative total of 29, 077 entries by the end of 1990.

Contrary to previous study [18], the study showed that the proportion of internationally co-authored articles in Nigeria's HIV/AIDS publications remained high during the study period. Some bibliometricians reported that 15.6% of the records in the SCI database in 2000 were internationally co-authored [19]. This and a previous study [18] found that articles with international collaboration appeared in journals with higher impact factors and received more citations. However, the causality was uncertain. A possible explanation for this could be that researcher sought foreign involvement only for better themes that were of broader interest and suitable for journals with higher impact factors. Furthermore, Roberts [20] argue that part of the explanation for the higher impact factor is probably that only the strongest researchers will have the resources and motivation to overcome the difficulties of collaboration over a distance, and that the biggest and highest profile projects are more likely to be international.

It is important to note that United States institutions formed most of institutional contributors; result that is congruent with the finding of previous studies [16,18]. Echoing general observation made by Professor Sir Gareth that USA, as the world's biggest research economy, is the preferred partner for international research partnerships and makes a significant contribution to the leading edge performance of collaborating nations [20].

A high pattern of co-authorship was found. Over 85% of the articles were published in collaboration among two or more authors. This finding is consistent with an earlier study from Haiti [16]. The results also show that articles with more than four authors appeared in journals with higher impact factors and received more citations; this finding is intriguing and would need benefit from further exploration. About one third of the records indexed to public, environment and occupational health subject category; and only 1.9% were indexed to education and educational research. This is consistent with a previous report [16]. This finding suggests the type of research that is needed. More research on prevention and control are needed to guide evidence-based HIV prevention programs.

### Study limitations

Potential limitations of the study are related to the database used to retrieve articles. ISI databases do not represent all scientific and biomedical journals published. Many articles of importance appear in journals other than those indexed in ISI database. Other limitations include the incorrect or multiple citations of the subject category that was generally used in compilation of the annual Journal Citation Reports^® ^presented in each ISI record. One journal with its articles might be indexed with several subject categories. Absolute country counting was adopted in accrediting an article to countries and might have overlooked the indirect collaboration relationships. Research collaboration might exist in several forms and levels: individual, group, department, institution, sector, and nation [21]. As noted by Chen et al [18], adopting a single impact factor value to judge a journal could create some discrepancies. This study used 2006 Journal Citation Reports. The 2006 JCR reported the performance of journals published in 2004 and 2005. However, the articles that were retrieved from the Web of Science were distributed between 1987 and 2006. The journal impact factor changes from year to year. The major limitation in analyzing the institutional research performance in Nigeria was the number of research staff in each institution was unknown. Therefore, the study could not examine the potential confounding effect of number of research staff on HIV research production.

## Conclusion

In summary, Nigeria achieved a significant increase in the number of SCI publications and collaborations in HIV literature from 1987 to 2005. As noted by Katz et al [21], research collaboration is good and should be encouraged. Yet there exists opportunity for improvement in international collaboration. There is need to challenge the status, scientists from Nigeria should forge multiple collaborations beyond historical, political, and cultural lines to share knowledge and expertise on HIV/AIDS. Research has helped to quantify HIV associated morbidity and mortality, identified strategies to improve health of people living with HIV/AIDS, and shown the effectiveness of HIV/AIDS preventive interventions. Furthermore, comparison analyses of HIV/AIDS literature production in Nigeria with other countries from sub-Saharan need to be conducted to obtain a more complete regional picture of the situation. These analyses will provide further support to AIDS researchers, health policy analyst, and librarians or information officers in the sub-Saharan Africa.

## Competing interests

The author(s) declare that they have no competing interests.

## Authors' contributions

OAU conceived the study, extracted the data, did the analyses and interpretation, and wrote the first and final draft of the manuscript.

## Pre-publication history

The pre-publication history for this paper can be accessed here:


